# Susceptibility of brown adipocytes to pro-inflammatory cytokine toxicity and reactive oxygen species

**DOI:** 10.1042/BSR20150193

**Published:** 2016-03-03

**Authors:** Lars Rebiger, Sigurd Lenzen, Ilir Mehmeti

**Affiliations:** * Institute of Clinical Biochemistry, Hannover Medical School, 30623 Hannover, Germany

**Keywords:** antioxidative enzymes, brown adipocyte cell line, brown adipose tissue, pro-inflammatory cytokines, reactive oxygen species, UCP-1

## Abstract

Brown adipose tissue (BAT) cells have a very high oxidative capacity. On the other hand, in obesity and obesity-related diabetes, levels of pro-inflammatory cytokines are elevated, which might promote BAT dysfunction and consequently impair carbohydrate metabolism and thereby exacerbate cellular dysfunction and promote diabetes progression. Therefore, the antioxidative enzyme status of a brown adipocyte cell line and its susceptibility towards pro-inflammatory cytokines, which participate in the pathogenesis of diabetes, and reactive oxygen species (ROS) were analysed. Mature brown adipocytes exhibited significantly higher levels of expression of mitochondrially and peroxisomally located antioxidative enzymes compared with non-differentiated brown adipocytes. Pro-inflammatory cytokines induced a significant decrease in the viability of differentiated brown adipocytes, which was accompanied by a massive ROS production and down-regulation of BAT-specific markers, such as uncoupling protein 1 (UCP-1) and β-Klotho. Taken together, the results strongly indicate that pro-inflammatory cytokines cause brown adipocyte dysfunction and death through suppression of BAT-specific proteins, especially of UCP-1 and β-Klotho, and consequently increased oxidative stress.

## Abstract (precis)

Pro-inflammatory cytokine-induced brown adipocyte dysfunction and consecutive cell death is mediated by suppression of the mitochondrial uncoupling protein 1 and concomitant generation of reactive oxygen species.

## INTRODUCTION

Obesity is a major predisposing factor for many diseases, including arteriosclerosis, hypertension and type 2 diabetes mellitus [1]. Elevated levels of circulating free fatty acids (FFAs) due to obesity cause peripheral insulin resistance and progressive β-cell loss via ER stress and generation of reactive oxygen species (ROS) [2,3]. Brown adipose tissue (BAT) is a highly active metabolic organ, which is able to accommodate high amounts of glucose and lipids from the circulation and therefore mediates energy dissipation and glucose clearance. Thus, BAT can contribute to an improved glucose and lipid homoeostasis, and concomitantly weight loss [4,5]. Several studies provide evidence that rodents with higher amounts of BAT gain less weight, are more sensitive to insulin, have lower FFA levels in the circulation, and are protected against type 2 diabetes and other obesity-related metabolic disorders [4,6,7]. In addition, more recently it has been shown that stimulation of BAT activation in humans is accompanied by significantly increased resting energy expenditure, whole-body glucose disposal, plasma glucose oxidation and insulin sensitivity [8].

BAT as a thermogenic tissue is characterized by a large number of mitochondria, lipid droplets and an extremely high oxidative capacity. Upon stimulation, BAT induces thermogenesis by dissipating large amounts of chemical energy as heat. This process is mediated by the BAT-specific mitochondrial uncoupling protein 1 (UCP-1), which facilitates proton leakage from the intramembrane space into the mitochondrial matrix independent of ATP production [6,9,10]. Previous studies suggested that augmented uncoupling of oxidative phosphorylation and consequently increased energy expenditure is accompanied by generation of large quantities of mitochondrial ROS [11,12]. However, recent observations indicate that UCP-1-mediated uncoupling of oxidative phosphorylation prevents ROS production and oxidative damage in BAT [13,14]. Although the function of activated UCP-1 in mitigating ROS is still disputed, it is now well recognized that BAT is an active endocrine organ that secretes numerous adipokines, which have both endocrine and paracrine effects [10,15]. It has been proposed that secreted pro-inflammatory cytokines such as interleukin 1 and 6 are involved in lipolysis and secretion of FFA, thereby contributing to insulin resistance [3].

Although BAT has been considered a unique therapeutic target in the prevention of obesity and type 2 diabetes [16,17], the relationship between ROS and pro-inflammatory cytokines and their influence on BAT viability is still unknown. Therefore we characterized the antioxidative defense status of a brown adipocyte cell line during differentiation and thereafter determined its antioxidative capacity in response to intramitochondrial ROS generators and pro-inflammatory cytokines as well as their effects on the expression of adipocyte-specific genes.

## MATERIALS AND METHODS

### Cell culture and differentiation

The murine non-differentiated brown adipocyte cell line [18] (kindly provided by Dr A. Whittle, Institute of Metabolic Science, University of Cambridge, UK) was cultured in Dulbecco's Modified Eagle's Medium (DMEM) supplemented with 25 mM glucose, 10% fetal calf serum (FCS), 1% glutamine, 1% penicillin and 1% streptomycin in a humidified atmosphere at 37°C and 5% CO_2_. Differentiation of the brown adipocyte cell line into mature brown adipocytes was conducted as described [18,19]. The density of non-differentiated brown adipocytes depending on the further experimentation was determined by the Cellometer Auto T4 counter (Nexcelom Bioscience).

### Oil Red O (ORO) staining

Non-differentiated brown adipocytes were seeded overnight at a density of 1.5×10^4^ per well on 35 mm glass bottom culture dishes (Glass No. 0, MatTek Corporation) and differentiated into mature brown adipocytes. At days 1, 3, 6 and 9 of differentiation the brown adipocytes were washed twice with PBS and fixed with 4% paraformaldehyde at 4°C overnight. Thereafter fixed brown adipocytes were washed three times with PBS and stained with 3.7 mM freshly prepared Oil Red O (ORO) solution at room temperature. After a 20 min ORO incubation, brown adipocytes were washed three times with PBS and examined under an Olympus IX81 inverted microscope. Microscopic images were post-processed using the Olympus-specific software Xcellence (Olympus).

### Quantitative real-time PCR (qRT-PCR)

Total RNA from brown adipocytes at differentiation days 1, 3, 6 and 9 was isolated using the RNeasy Mini Kit (Qiagen) before and after incubation with pro-inflammatory cytokines. Isolated RNA was quantified and analysed by NanoDrop 1000 (Thermo Scientific). Two micrograms of RNA were reverse transcribed into cDNA by using random hexamer primers. For quantitative real-time PCR (qRT-PCR), QuantiTect SYBR Green technology (Qiagen) and the ViiA™ 7 Real-Time PCR System (Life Technologies) were used. Samples were incubated at 50°C for 5 min followed by a 10 min denaturation step at 95°C. Following this initial denaturation, the 40 PCR cycles comprised a denaturation step at 95°C for 30 s, an annealing step at 60°C for 30 s and an extension step at 72°C for 30 s. The optimal PCR reaction parameters were defined empirically and the purity and specificity of the amplified PCR product in each experiment was verified by melting curves. Primers were designed to have annealing temperatures of 60°C and to provide amplicons between 79 and 190 base pairs (Supplementary Table S1). All measurements were performed in triplicate and normalized to the housekeeping genes β-actin, peptidylprolyl isomerase A (PPIA) and α-tubulin using the QBasePLUS data analysis software (Biogazelle).

### Western blot analysis

For protein analysis non-differentiated brown adipocytes cells were plated at a density of 4×10^5^ cells/10 cm dish and differentiated according to the differentiation protocol. At differentiation days 1, 3, 6 and 9, cells were prepared in RIPA Buffer (Sigma–Aldrich) supplemented with protease inhibitor cocktail (Roche Diagnostics). Protein concentration was determined by the BCA (bicinchoninic acid) assay (Pierce). Twenty micrograms of isolated protein was subjected to SDS-PAGE (10% for catalase, 12% for GPx1, MnSOD and CuZnSOD) and transferred to PVDF membranes (GE Healthcare Life Sciences). Five percent non-fat dry milk was used to block nonspecific binding sites of the membranes at 4°C overnight. After washing three times with wash buffer (PBS, 0.1% Tween), membranes were incubated with specific antibodies against CuZnSOD (1:3000), MnSOD (1:3000), GPx1 (1:7500, kind gifts from Dr K. Dobashi, Japan) and catalase (1:500, F-17, Santa Cruz, Dallas, USA) for 4 h at room temperature or overnight at 4°C. Thereafter, the membranes were incubated with specific peroxidase-labelled anti-rabbit secondary antibody (1:20000, Code No.: 711-035-152, Dianova) at room temperature for 1 h. Hybridized antibodies were visualized by the chemiluminescence-based ECL detection system (Amersham Bioscience). Subsequently, the membranes were stripped (ReBlot Plus Strong Solution, Millipore, Germany), blocked and re-incubated with primary antibody against β-actin (1:250, C-11, Santa Cruz, Dallas, USA), followed by incubation with peroxidase-conjugated anti-goat secondary antibody (1:20000, Code No.: 705-035-147, Dianova) at room temperature for 1 h. The protein bands were visualized by chemiluminescence as described above. Band intensity was quantified through densitometry with Gel-Pro Analyzer 6.0 (Media Cybernetics).

### Determination of cell viability

Non-differentiated brown adipocytes were seeded at a density of 3000 cells per well on to 96-well plates for full differentiation into brown adipocytes before they were exposed to pro-inflammatory cytokines, H_2_O_2_ or menadione. Differentiated brown adipocytes were incubated with differentiation medium (control) or with different pro-inflammatory cytokines, IL-1β (600 U/ml; 48 ng/ml) alone, a cytokine mixture (IL-1β 60 U/ml; 4.8 ng/ml, TNF-α 185 U/ml; 9.3 ng/ml, IFN-γ 14 U/ml; 11.2 ng/ml), 10× TNF-α (1850 U/ml; 93 ng/ml) or 10× IFN-γ (140 U/ml; 112 ng/ml) for 72 h (cytokines were purchased from PromoCell). The choice of cytokine concentration and incubation period was based on previous studies from our group regarding their cytotoxic effects on β-cell viability [20,21]. Time course pre-experiments with the brown adipocyte cell line treated with the aforementioned cytokines revealed that incubation times shorter than 72 h induce no significant cytotoxic effect (Supplementary Figure S1), confirming earlier observations in other cell types [20,21]. Therefore an incubation period of 72 h was adapted for cytotoxicity analyses. Importantly, culturing of differentiated adipocytes in medium under control conditions for periods up to 72 h had no negative effect on cell viability. Since we only performed time-dependent but no concentration-dependent analysis, we cannot fully exclude that higher cytokine concentrations may be more toxic. The ROS generator menadione was freshly dissolved and cells were incubated in a concentration range between 0 and 50 μM in differentiation medium for 2 h, whereas the exposure of mature adipocytes to H_2_O_2_ in a concentration range from 0 to 2000 μM occurred in Hepes (20 mmol/l)-supplemented Krebs–Ringer bicarbonate medium with 5 mmol/l glucose. Thereafter the menadione or H_2_O_2_ containing medium was replaced by fresh differentiation medium and cultured overnight. Cell viability was determined by a microplate-based MTT assay using (3-(4,5-dimethylthiazol-2-yl)-2,5-diphenyltetrazolium bromide) from Sigma–Aldrich as described [22].

### Determination of ROS production

Non-differentiated brown adipocytes were seeded at a density of 3000 cells per well on to 96-well plates and fully differentiated into brown adipocytes. At differentiation day 9, brown adipocytes were incubated with 10 μM H2DCFDA (Life Technologies GmbH) for 40 min and thereafter treated with differentiation medium (control), IL-1β (600 U/ml) alone, a cytokine mixture (60 U/ml IL-1β, 185 U/ml TNF-α, 14 U/ml IFN-γ), 10× TNF-α (1850 U/ml) or 10× IFN-γ (140 U/ml) for 72 h. ROS generation was measured at 480/520 nm excitation/emission using a Victor^2^ 1420 Multilabel Counter (PerkinElmer) as described [22].

### Statistical analysis

All data are presented as means±S.E.M. Graphs and statistical analyses were made with GraphPad Prism 5 (GraphPad Software). Statistical significance was determined using one way ANOVA prior to Dunnett's Multiple Comparison Test, as appropriate. Only *P* values lower than 0.05 (*P*<0.05) were considered as statistically significant.

## RESULTS

### Adipogenic differentiation of the murine brown preadipocyte cell line

**Figure 1:**
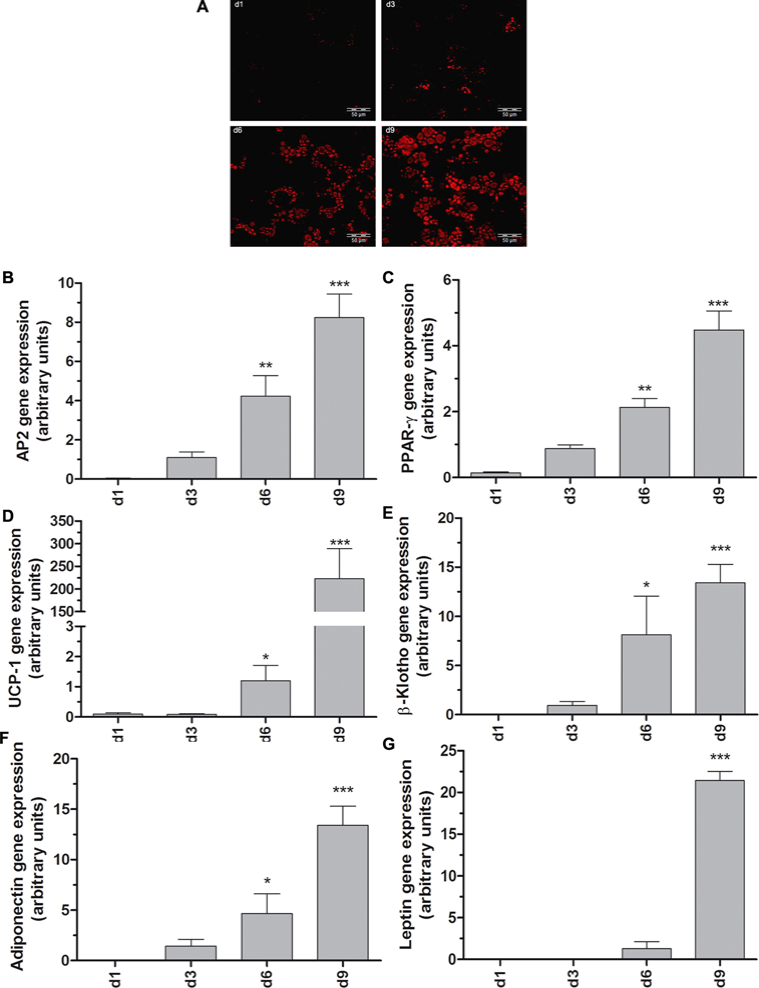
Adipogenic differentiation of the murine non-differentiated brown adipocyte cell line Non-differentiated brown adipocytes were seeded at a density of 1.5×10^4^ per well on 35 mm glass bottom culture dishes and differentiated according to the differentiation protocol. At differentiation days 1, 3, 6 and 9 (**A**) cells were fixed with 4% paraformaldehyde at 4°C. Cells were then washed with PBS and stained with 3.7 mM freshly prepared ORO solution at room temperature. After 20 min ORO incubation, cells were washed three times with PBS and examined with an Olympus IX81 inverted microscope. In addition non-differentiated brown adipocyte cells were seeded at a density of 4×10^5^ cells on 10 cm dishes and differentiated according to the differentiation protocol. At differentiation days 1, 3, 6 and 9 RNA was isolated and reverse transcribed into cDNA. Gene expression of aP2 (**B**), PPAR-γ (**C**), UCP-1 (**D**), β-Klotho (**E**), adiponectin (**F**) and leptin (**G**) was measured by qRT-PCR and normalized to the housekeeping genes β-actin, PPIA and α-tubulin. Data are means±S.E.M. of *n*=6. *P<0.05, **P<0.01, ***P<0.001 compared with cells at differentiation day 1 (Dunnett's Multiple Comparison Test).

Brown adipocytes harbour a large number of mitochondria, lipid droplets, and have an extremely high oxidative capacity [5,23]. To determine the antioxidative status and the susceptibility of BAT towards toxicity of pro-inflammatory cytokines and ROS generators, the murine non-differentiated brown adipocytes were induced into adipogenesis. Adipogenic differentiation was evaluated by ORO staining, a selective marker for lipid droplets. As shown in Figure 1(A), non-differentiated brown adipocytes were negative for ORO staining at day 1, indicating a lack of lipid droplets. After a 3 day induction of adipogenic differentiation lipid droplets were detectable with the ORO staining whose density markedly increased at day 6. At day 9 more than 90% of brown preadipocytes exhibited the morphology of mature adipocytes, as characterized by a large quantity of lipid droplets (Figure 1A, d9). According to the morphological changes, the gene expression of aP2 and PPAR-γ was significantly increased in differentiated brown adipocytes (days 6 and 9) compared with non-differentiated cells (Figures 1B and 1C).

Besides morphological changes, adipogenic differentiation comprises the modulation of the expression profiles of specific genes that determine the adipocyte character. Therefore, the gene expression of the brown adipocyte-specific markers UCP-1 and β-Klotho and also of adiponectin and leptin was quantified in differentiating cells. As shown in Figure 1(D) the UCP-1 gene expression level was 7.5-fold at day 6 and even 1500-fold higher at day 9 compared with differentiation day 1. Consistently, β-Klotho, an important auxiliary protein involved in glucose uptake in adipose tissue [24], showed at day 6 a 7-fold and at day 9 a 12.5-fold higher expression level as compared with day 1 (Figure 1E). The expression level of adiponectin was significantly increased at day 6 and achieved a maximum at day 9, whereas leptin expression was massively induced only at day 9 post induction (Figures 1F and 1G).

### Quantification of superoxide radical- and H_2_O_2_-inactivating enzyme expression during adipogenic differentiation

**Figure 2:**
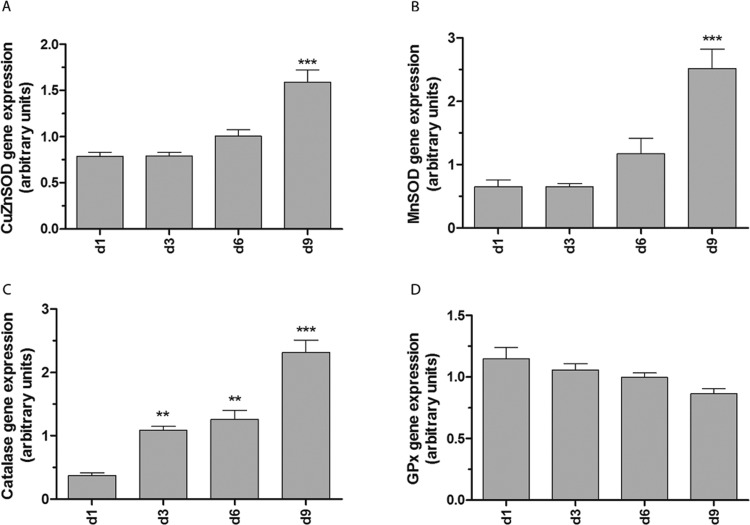
Gene expression of superoxide radical- and H_2_O_2_-inactivating enzymes during the differentiation of brown adipocytes Non-differentiated brown adipocytes were seeded at a density of 4×10^5^ cells on 10 cm dishes and differentiated according to the differentiation protocol. At differentiation days 1, 3, 6 and 9 RNA was isolated and reverse transcribed into cDNA. Gene expression of CuZnSOD (**A**), MnSOD (**B**), catalase (**C**) and GPx1 (**D**) was measured by qRT-PCR and normalized to the housekeeping genes β-actin, PPIA and α-tubulin. Data are means±S.E.M. of *n*=6. **P<0.01, ***P<0.001 compared with cells at differentiation day 1 (Dunnett's Multiple Comparison Test).

**Figure 3:**
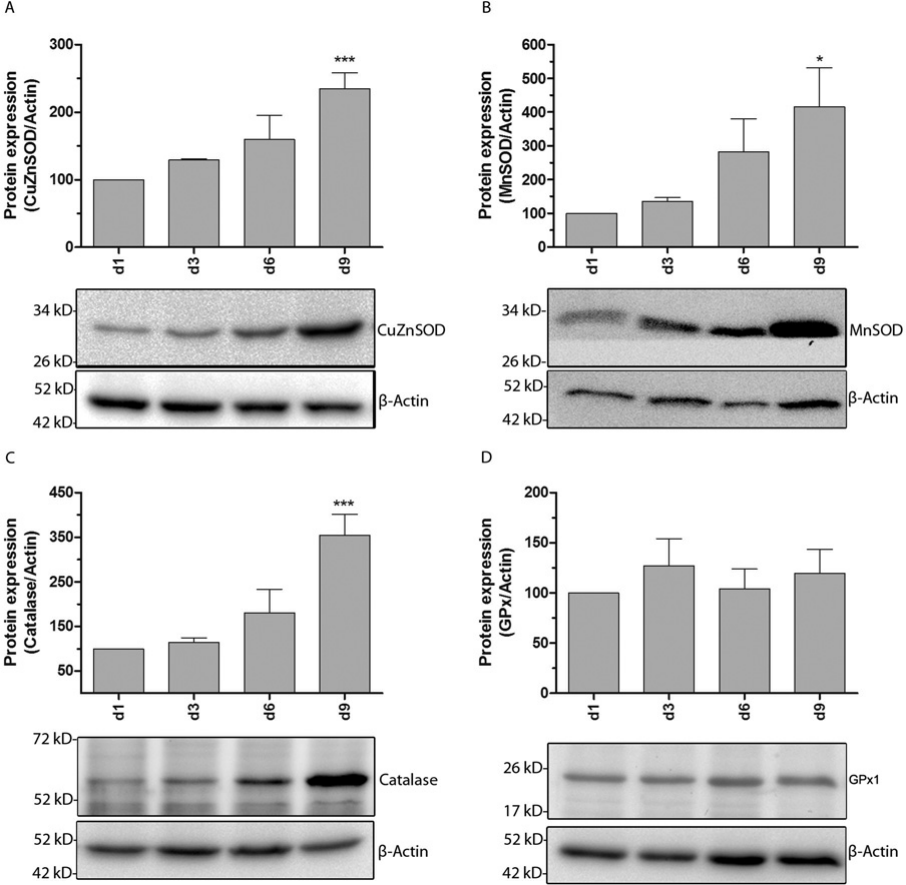
Protein expression of superoxide radical- and H_2_O_2_-inactivating enzymes during the differentiation of brown adipocytes Non-differentiated brown adipocytes were seeded at a density of 4×10^5^ cells on 10 cm dishes and differentiated according to the differentiation protocol. At differentiation days 1, 3, 6 and 9 cells were trypsinized and total protein was collected. Twenty micrograms of isolated protein was subjected to SDS-PAGE and immunoblotted using specific antibodies against CuZnSOD (**A**), MnSOD (**B**), Catalase (**C**) and GPx1 (**D**). Quantification of all investigated proteins is shown in relation to the housekeeping protein β-actin after reblotting. The protein expression at day 1 was set as 100%. Data are means±S.E.M. of *n*=6. *P<0.05, ***P<0.001 compared with cells at differentiation day 1 (Dunnett's Multiple Comparison Test).

To determine whether the antioxidative status of brown adipocytes changed during adipogenic differentiation, the expression of superoxide-inactivating enzymes CuZnSOD and MnSOD and of H_2_O_2_-detoxifying enzymes catalase and glutathione peroxidase-1 (GPx1) was characterized at days 1, 3, 6 and 9 post induction of adipogenesis. The superoxide-inactivating enzymes CuZnSOD (cytosolically located) and MnSOD (mitochondrially located) showed the highest expression level at day 9 when compared with day 1 (Figures 2A and 2B), whereas the expression of H_2_O_2_-detoxifying catalase was already significantly up-regulated at day 3 with the highest expression level also at day 9 (Figure 2C). However, interestingly, the GPx1 expression was not affected during differentiation. Consistent with the observed changes in mRNA levels, immunoblot analyses revealed a very high correlation with the gene expression data (Figure 3). But the observed induction of catalase gene expression at days 3 and 6 could not be verified at the protein level (Figure 3C). Moreover, the expression of all six peroxiredoxins (Prx1–6), which are able to consume H_2_O_2_, was quantified accordingly. As shown in Supplementary Figures S2(C) and S2(E) only the gene expression of the mitochondrially located Prx3 and Prx5 was markedly induced at day 9 post induction compared with day 1, whereas the expression of all other investigated peroxiredoxins (Supplementary Figures S2A–S2F) was not affected due to the differentiating process.

### Effects of pro-inflammatory cytokines on cell viability and ROS generation in mature brown adipocytes

**Figure 4:**
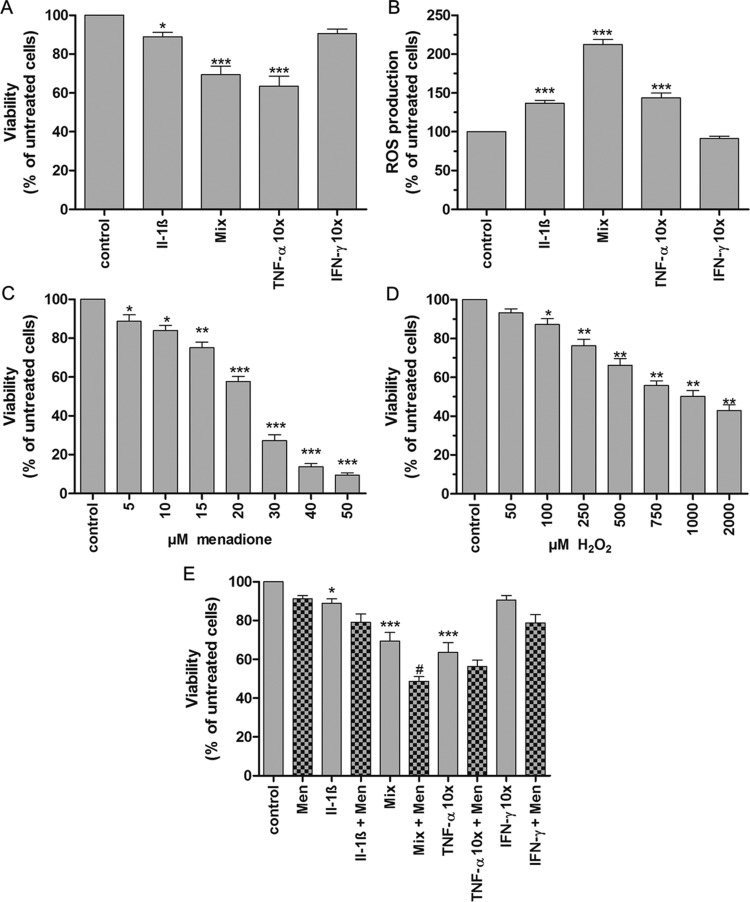
Effects of pro-inflammatory cytokines, menadione and exogenously added H_2_O_2_ on cell viability and cytokine-mediated ROS generation in differentiated brown adipocytes Three thousand non-differentiated brown adipocytes per well were seeded on to 96-well plates and differentiated according to the differentiation protocol. (**A**) Differentiated brown adipocytes were incubated for 72 h with differentiation medium (control), IL-1β (600 U/ml), a cytokine mixture (60 U/ml IL-1β, 185 U/ml TNF-α, 14 U/ml IFN-γ), 10× TNF-α (1850 U/ml) or 10× IFN-γ (140 U/ml). Cell viability was determined using the MTT assay and results are expressed as percentage relative to cells incubated under control conditions. (**B**) Differentiated brown adipocytes were incubated with DCF for 40 min and afterwards treated with cytokines as described in (**A**). After 72 h ROS production was measured and expressed as a percentage of control cells. (**C**) For 2 h differentiated brown adipocytes were treated with menadione (0, 5, 10, 15, 20, 30, 40, 50 μM) or (**D**) H_2_O_2_ (0, 50, 100, 250, 500, 750, 1000, 2000 μM). Thereafter the menadione or H_2_O_2_ containing medium was replaced overnight with differentiation medium. After 24 h cell viability was determined using the MTT assay. (**E**) Differentiated brown adipocytes were pretreated with menadione (5 μM) for 2 h and thereafter exposed to cytokines as indicated in (A). The MTT results are presented as a percentage relative to cells incubated under control conditions. Data are means±S.E.M. of *n*=14–16 (**A** and **C**) or *n*=6–8 (**B**, **D** and **E**). *P<0.05, **P<0.01, ***P<0.001 compared with control cells (Dunnett's Multiple Comparison Test); ^#^*P*<0.05 compared with cells incubated under the same conditions plus menadione (*t* test, unpaired, two-tailed).

In order to determine the susceptibility of brown adipocytes towards cytokine-induced toxicity, fully differentiated brown adipocytes were treated with the pro-inflammatory cytokine IL-1β alone (600 U/ml), a cytokine mixture consisting of IL-1β (60 U/ml), TNF-α (185 U/ml) and IFN-γ (14 U/ml), and with high concentrations of TNF-α (1850 U/ml) or IFN-γ (140 U/ml) for 72 h. Exposure of fully differentiated brown adipocytes to IL-1β alone resulted in a moderate (15%) reduction in cell viability compared with untreated cells. However, the cytokine mixture caused a significant decrease (*P*<0.05) in cell viability of 30% (Figure 4A). Treatment of brown adipocytes with TNF-α alone at high concentrations (1850 U/ml) decreased the cell viability by 36.5% (*P*<0.001). By contrast, IFN-γ at high concentrations (140 U/ml) did not significantly affect the brown adipocyte viability. Thus, TNF-α was the most toxic cytokine (Figure 4A). Pro-inflammatory cytokines, especially TNF-α are known to induce mitochondrial ROS generation in other cell types [25,26]. Therefore the effects of the aforementioned cytokines on ROS generation in fully differentiated brown adipocytes were investigated. As shown in Figure 4(B) the exposure of fully differentiated brown adipocytes to IL-1β and TNF-α alone as well as to the cytokine mixture led to a significantly increased (*P*<0.001) overall ROS production with the cytokine mixture having the strongest effect (IL-1β, TNF-α 50% each, cytokine mixture ∼110%, compared with untreated cells). Notably, in analogy to the cell viability data, IFN-γ did also not induce ROS generation (Figure 4B). To test whether brown adipocytes are susceptible to ROS toxicity, differentiated brown adipocytes were treated with increasing concentrations of menadione (0, 5, 10, 15, 20, 30, 40, 50 μM), an intramitochondrial ROS generator, or of H_2_O_2_ (0, 50, 100, 250, 500, 750, 1000, 2000 μM). After a 2 h exposure to the chemical menadione a concentration-dependent loss of cell viability with a maximum 90% decrease at 50 μM was observed (Figure 4C). Exogenously added H_2_O_2_ also caused a significant decrease in brown adipocyte viability in a concentration-dependent manner (Figure 4D). Thus, brown adipocytes are highly susceptible towards ROS-mediated toxicity. In order to evaluate whether the deleterious effects of pro-inflammatory cytokines in brown adipocytes result from ROS-induced cell damage, brown adipocytes were pretreated with 5 μM menadione, a concentration shown in concentration–response experiments to induce a mild toxicity, followed by cytokine exposure. As shown in Figure 4(E) preconditioning brown adipocytes with the mitochondrial ROS generator menadione exacerbated the toxicity of IL-1β and TNF-α alone as well as the toxicity of the cytokine mixture, strongly indicating that ROS of mitochondrial origin are involved in pro-inflammatory cytokine-mediated toxicity.

### Effects of pro-inflammatory cytokines on adipocyte-specific gene expression

**Figure 5:**
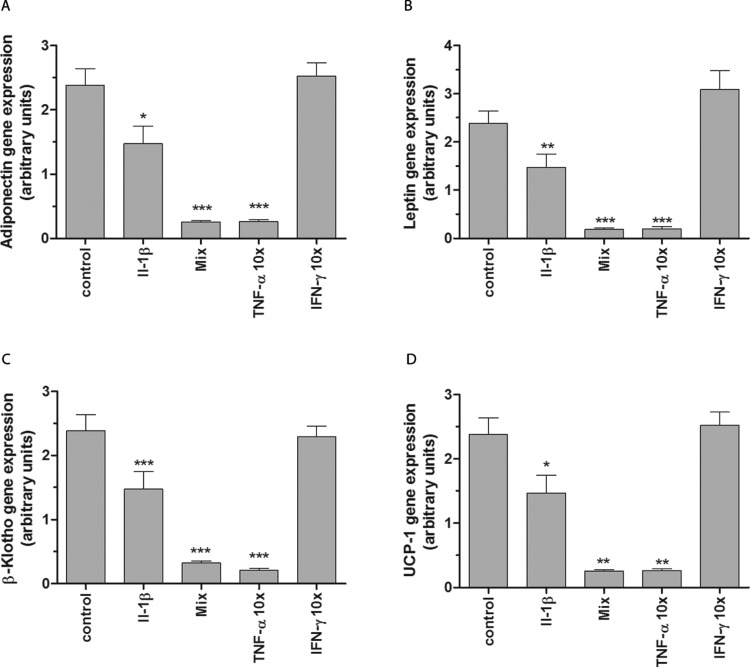
Effects of pro-inflammatory cytokines on adipocyte-specific gene expression Non-differentiated brown adipocytes were seeded at a density of 4×10^5^ cells on 10 cm dishes and differentiated according to the differentiation protocol. Differentiated brown adipocytes were incubated with differentiation medium (control), IL-1β (600 U/ml), a cytokine mixture (60 U/ml IL-1β, 185 U/ml TNF-α, 14 U/ml IFN-γ), 10× TNF-α (1850 U/ml) or 10× IFN-γ (140 U/ml). After 24 h total RNA was isolated and reverse transcribed into cDNA. Gene expression of adiponectin (**A**), leptin (**B**), β-Klotho (**C**) and UCP-1 (**D**) was measured by qRT-PCR and normalized to the housekeeping genes β-actin, PPIA and α-tubulin. Data are means±S.E.M. of *n*=8. *P<0.05, **P<0.01, ***P<0.001 compared with control cells (Dunnett's Multiple Comparison Test).

Having shown that pro-inflammatory cytokines, except for IFN-γ, decreased cell viability and induced ROS production, we next examined the effects of these pro-inflammatory cytokines on the gene expression of adiponectin and leptin and also on the expression of BAT-specific markers (UCP-1 and β-Klotho). Treatment of fully differentiated brown adipocyte cells with IL-1β alone led to a slight but statistically significant suppression of adiponectin (Figure 5A), leptin (Figure 5B), β-Klotho (Figure 5C) and UCP-1 (Figure 5D) gene expression. However, high concentrations of TNF-a (1850 U/ml) alone and a cytokine mixture nearly completely inhibited the gene expression of adiponectin, leptin, β-Klotho and UCP-1 (Figures 5A–5D). Again, IFN-γ had no effect on transcription of the investigated BAT markers (Figure 5).

## DISCUSSION

The prevalence of obesity and obesity-related diseases has reached epidemic levels and is still increasing worldwide. In particular type 2 diabetes is strongly associated with weight gain [15,27]. To combat obesity and its comorbidities, BAT with its unique anti-obesity properties has currently moved into the focus of new therapeutic strategies [1,5,28]. Several studies provide evidence that an increase in BAT, either by transplantation or activation, plays a significant beneficial role in whole-body energy expenditure, carbohydrate homoeostasis, and insulin sensitivity in both rodents [4,6,7] and humans [8]. On the other hand, there is increasing evidence to suggest that changes in BAT activity are frequently associated not only with the increased glucose disposal and lipid uptake but also with the generation and secretion of a wide range of molecules, such as pro-inflammatory cytokines [10,15] and ROS [11,12]. These mediators could cause BAT dysfunction and consequently impair carbohydrate metabolism, thereby aggravating cellular dysfunction in obesity-related diseases. Therefore we sought to determine the antioxidative defense status and the susceptibility of a brown adipocyte cell line towards toxicity of pro-inflammatory cytokines and ROS. In the current study we showed that the expression of both the superoxide-inactivating enzymes CuZnSOD and MnSOD and of H_2_O_2_-detoxifying enzymes, especially of peroxisomal catalase and mitochondrially located peroxiredoxins (Prx3 and Prx5) was significantly increased, whereas interestingly that of the cytosolically located H_2_O_2_-detoxifying enzymes (peroxiredoxin 1, 2 and 6) was not affected during adipogenic differentiation. In addition we demonstrated that pro-inflammatory cytokines induced a significant reduction in the expression of essential adipose tissue markers such as UCP-1, which was accompanied by a massive increase in ROS production and a subsequent loss of viability of brown adipocytes.

BAT with its high content of mitochondria and elevated UCP-1 expression is capable of significantly increasing total energy expenditure by predominately consuming FFAs and also glucose as substrates. To meet increased metabolic demands of stimulated BAT, lipolysis and subsequently β-oxidation of released FFAs are markedly increased [4]. The fact that the long and very long FFAs are β-oxidized in peroxisomes and also in mitochondria [2,29], inevitably generating H_2_O_2_ as a by-product, the observed enhanced antioxidative capacity in mitochondria and peroxisomes strongly indicates an increased need of BAT for protection against oxidative injury in these organelles. Indeed, despite the presence of scavenging enzymes, exposure of brown adipocyte cells to the intramitochondrial ROS generator menadione and to exogenously added H_2_O_2_ resulted in a strong reduction in cellular viability.

Accumulating evidence suggests that BAT is not only specialized in glucose disposal and lipid uptake, but is also an endocrine organ capable of expressing and secreting endocrine mediators with auto- and paracrine effects [10,15]. BAT-derived IL-1α and in particular IL-6 are critical pro-inflammatory cytokines in the induction of insulin resistance [30,31] and activation of the immune system [32,33]. Conversely, inflammation is considered as a critical contributing factor in pancreatic β-cell dysfunction and death [34]. A chronic increase in inflammatory mediators, such as IL-1β, TNF-α and IL-6, as detected in obese type 2 diabetic subjects [35,36], might affect not only pancreatic β-cells but also BAT. In particular, an increase in IL-1β has been shown to promote pancreatic β-cell destruction, which in combination with TNF-α and IL-6 aggravates the extent of β-cell apoptosis in a synergistic way [37,38]. In the present study we demonstrated that β-cell-toxic pro-inflammatory cytokines, especially the cytokine mixture and TNF-α, had a significant toxic effect on brown adipocytes. This cytokine-mediated brown adipocyte toxicity was accompanied by a significant reduction in the expression of the most abundant BAT protein UCP-1 and concomitantly ROS generation. These results are in agreement with earlier reports showing that TNF-α affects BAT thermogenesis by down-regulation of UCP-1 expression [39–41], and that induction of UCP-1 expression prevents ROS production and oxidative damage in BAT [13,14,42]. Recent evidence suggests that uncoupling of the mitochondrial membrane potential by UCP-1 reduces the ROS generation in the mitochondria of tenrecs, indicating that UCP-1 is an efficient evolutionary conserved mitochondrial ROS scavenger [43]. Moreover, the gene expression of adiponectin, which is known to prevent inflammation [44] and ROS formation [45,46] and also that of β-Klotho, a crucial co-factor mediating FGF21 (fibroblast growth factor 21) function [24], was significantly impaired in response to pro-inflammatory cytokines. Thus, pro-inflammatory cytokines cause death of brown adipocytes by suppression of proteins with antioxidative properties, e.g. UCP-1, and consequently a massive increase in ROS formation, which might cause mitochondrial oxidative damage and cell death as reported earlier in other cell types [47]. This assumption is supported by the observation that preconditioning brown adipocyte cells with non-lethal concentrations of the intramitochondrial ROS generator menadione exacerbated the cytotoxic effect of cytokines.

Overall, the present results provide evidence that brown adipocyte cells are highly sensitive to pro-inflammatory cytokines and intramitochondrially generated ROS. Moreover, we could show that pro-inflammatory cytokines cause a significant reduction in the most prominent BAT markers (UCP-1 and β-Klotho) and consequently increase ROS generation, which might overwhelm the antioxidative capacity of BAT, causing oxidative stress and ultimately BAT damage.

## Abbreviations

BAT: brown adipose tissue
FFA: free fatty acid
ORO: Oil red O
PPIA: peptidylprolyl isomerase A
qRT-PCR: quantitative real-time PCR
ROS: reactive oxygen species
UCP-1: uncoupling protein 1
